# Cybercrime and Artificial Intelligence. An overview of the work of international organizations on criminal justice and the international applicable instruments

**DOI:** 10.1007/s12027-022-00702-z

**Published:** 2022-02-22

**Authors:** Cristos Velasco

**Affiliations:** 1 Center for AI and Digital Policy (CAIDP), Washington (DC), USA; 2 DHBW Cooperative State University in Mannheim and Stuttgart, Stuttgart, Germany; 3 Mexico City, Mexico

**Keywords:** AI, Budapest Convention, CAHAI, Criminal justice, Cybercrime, Deepfakes, Istanbul Convention, Law enforcement, Lanzarote Convention

## Abstract

The purpose of this paper is to assess whether current international instruments to counter cybercrime may apply in the context of Artificial Intelligence (AI) technologies and to provide a short analysis of the ongoing policy initiatives of international organizations that would have a relevant impact in the law-making process in the field of cybercrime in the near future. This paper discusses the implications that AI policy making would bring to the administration of the criminal justice system to specifically counter cybercrimes. Current trends and uses of AI systems and applications to commit harmful and illegal conduct are analysed including deep fakes. The paper finalizes with a conclusion that offers an alternative to create effective policy responses to counter cybercrime committed through AI systems.

## Introduction

Undoubtedly, AI has brought enormous benefits and advantages to humanity in the last decade and this trend will likely continue in coming years since AI is gradually becoming part of the digital services that we use in our daily lives. Many governments around the world are considering the deployment of AI systems and applications to help them achieve their activities and more concretely to facilitate the identification and prediction of crime.^1^ Further, national security and intelligence agencies have also realized the potential of AI technologies to support and achieve national and public security objectives.

There are significant developments of AI technologies like the use of facial recognition in the criminal justice realm, the use of drones, lethal autonomous weapons and self-driving vehicles that when not properly configured or managed without proper oversight mechanisms in place have the potential to be used for disruptive purposes and harm individual’s rights and freedoms.

Currently, there is an ongoing discussion in international policy and legislative circles on the revision and improvement of the liability framework and threshold concerning AI systems and technologies,^2^ although due to the complexity of the topic and the different legal approaches around the world concerning civil liability, there will probably not be a consensus on a harmonized and uniformed response, at least not in the near future.

Further, AI and machine learning have the potential and offer the possibility to detect and respond to cyberattacks targeted to critical infrastructure sectors including water, energy and electricity supplies, as well as the correct management of cybersecurity solutions to help reduce and mitigate security risks.^3^ However, many complex challenges remain particularly for small and medium enterprises which continue to rely on limited budgets to improve their cybersecurity capabilities.

Due to the COVID-19 pandemic, a large part of the world’s connected population was confined. This situation made companies and individuals more dependent on the use of systems, technologies and applications based on AI to conduct their activities, including remote work, distance learning, online payments or simply having access to more entertainment options like streaming and video on demand services. Unfortunately, this situation also led organized criminal groups to reconsider and re-organized their criminal activities in order to specifically target a number of stakeholders, including international organizations,^4^ research and health sector entities,^5^ supply chain companies^6^ and individuals. We have witnessed that organized criminal groups have largely improve their *CasS* (crime as a service) capabilities and turn their activities into higher financial profits with very small possibilities of being traced by law enforcement and brought to justice.

Through the use of AI technologies, cybercriminals have not only found a novel vehicle to leverage their unlawful activities, but particularly new opportunities to design and conduct attacks against governments, enterprises and individuals. Although, there is no sufficient evidence that criminal groups have a strong technical expertise in the management and manipulation of AI and machine learning systems for criminal purposes, it is true that said groups have realized its enormous potential for criminal and disruptive purposes.^7^ Further, organized criminal groups currently recruit and bring technical skilled hackers into their files to manipulate, exploit and abuse computer systems and to perpetrate attacks and conduct criminal activities 24/7 from practically anywhere in the world.^8^

## Current cybercrime trends

Current trends and statistics show that cybercriminals are relying more on the use of IoT to write and distribute malware and target ransomware attacks which are largely enhanced through AI technologies.^9^ This trend will likely continue as it is expected that more than 2.5 million devices will be fully connected online in the next 5 years including industrial devices and critical infrastructure operators which will make companies and consumers more vulnerable to cyberattacks.^10^

Furthermore, the discussion on bias and discrimination^11^ are also relevant debated aspects on AI policy in many international and policy making circles.^12^ The widespread use of technologies based on facial recognition systems,^13^ deserves further attention in the international policy arena because even when facial recognition may be very appealing for some governments to enhance aspects of public security and safety to prioritize national security activities, including terrorist activities, this technology may as well raises relevant and polemic issues concerning the protection of fundamental rights, including privacy and data protection under existing international treaties and conventions, topics that are currently being discussed in relevant international fora including the Council of Europe, the European Commission, the European Parliament^14^ and the OECD.

There is an ongoing global trend to promote misinformation with the support of AI technologies known as ‘bots’.^15^ Bots are mainly used to spread fake news and content throughout the internet and social networks and have the chilling effect of disinforming and misleading the population, particularly younger generations who cannot easily differentiate between legitimate sources of information and fake news. Further, the use of ‘bots’ have the potential to erode trust and question the credibility of the media and destabilize democratic and government institutions.

Although AI holds the prospect to enhance the analysis of big amounts of data to avoid the spread of misinformation in social networks,^16^ humans still face the challenge to check and verify the credibility of the sources, an activity which is usually conducted by content moderators of technology companies and media outlets without specific links to government spheres, a situation that has led relevant policy making institutions like the European Commission to implement comprehensive and broad sets of action to tackle the spread and impact of online misinformation.^17^

Another trend and technology widely used across many industries are deep fakes.^18^

The abuse and misuse of deepfakes has become a major concern in national politics^19^ and among law enforcement circles.^20^ Deepfakes have been used to impersonate politicians,^21^ celebrities and CEO’s of companies which may be used in combination with social engineering techniques and system automatization to perpetrate fraudulent criminal activities and cyberattacks. The use of deep fake technologies for malicious purposes is expanding rapidly and is currently being exploited by cybercriminals on a global scale. For example, in 2019, cybercriminals used AI voice generating software to impersonate the voice of a Chief Executive of an energy company based in the United Kingdom and were able to obtain $243,000 and distribute the transfers of the funds to bank accounts located in Mexico and other countries.^22^

Another relevant case occurred in January 2020 where criminals used deep voice technology to simulate the voice of the director of a transnational company. Through various calls with the branch manager of a bank based in the United Arab Emirates, criminals were able to steal $35 million that were deposited into several bank accounts, making the branch manager of the bank believe that the funds will be used for the acquisition of another company.^23^

The spoofing of voices and videos through deep fakes raise relevant and complex legal challenges for the investigation and prosecution of these crimes. First and foremost, many law enforcement authorities around the world do not yet have full capabilities and trained experts to secure evidence across borders, and often times the lack of legal frameworks particularly procedural measures in criminal law to order the preservation of digital evidence and investigate cybercrime represents another major obstacle. Second, since most of these attacks are usually orchestrated by well organized criminal groups located in different jurisdictions, there is the clear need for international cooperation, and in particular a close collaboration with global services providers to secure subscriber and traffic data, as well as to conduct more expedited investigations and law enforcement actions with other countries through the deployment of joint investigation teams in order to be able to trace and locate the suspects and follow the final destination of illicit funds.^24^ Cross-border cybercrime investigations are complex, lengthy, and do not always necessarily result in convictions of the perpetrators.

Further, cyberattacks based on AI systems is a growing trend identified by the European Cybercrime Centre (EC3) of EUROPOL in its *Internet Crime Threat Assessment Report 2020*. According to the EC3, the risks concerning the use of AI for criminal purposes need to be well understood in order to protect society against malicious actors. According to the EC3, “through AI, criminals may facilitate and improve their attacks by maximizing their opportunities for profit in a shorter period of time and create more innovative criminal business models, while reducing the possibility of being traced and identified by criminal justice authorities”.^25^

Further, the EC3 of EUROPOL recommends the development of further knowledge regarding the potential use of AI by criminals with a view to better anticipating possible malicious and criminal activities facilitated by AI, as well as to prevent, respond to, or mitigate the effects of such attacks in a more proactive manner and in close cooperation with industry and academia.^26^

## Strategic partnerships

Due to the complexities that the misuse and abuse of AI systems for criminal purposes entail for law enforcement agencies, key stakeholders are trying to promote the development of strategic partnerships between law enforcement, international organizations and the private sector to counter more effectively against the misuse and abuse of AI technologies for criminal purposes. For example, in November 2020, Trend Micro Research, the EC3 of EUROPOL and the Centre for Artificial Intelligence and Robotics of the UN Interregional Crime and Justice Research Institute (UNICRI) published the report: *Malicious Uses and Abuses of Artificial Intelligence*.^27^ This report contains an in-depth technical analysis of present and future malicious uses and abuses of AI and related technologies that drew from the outcomes of a workshop organized by EUROPOL, Trend Micro and UNICRI in March 2020. The report highlights relevant technical findings and contains examples of AI capabilities divided into “malicious AI uses” and “malicious AI abuses”. The report also sets forth future scenarios in areas like AI supported ransomware, AI detection systems, and developed a case study on deepfakes highlighting the development of major policies to counter it, as well as recommendations and considerations for further and future research.^28^

Strategic initiatives and more partnerships like the one mentioned above are further needed in the field of AI and cybercrime to ensure that relevant stakeholders particularly law enforcement authorities and the judiciary understand the complexities and dimensions of AI systems and start developing cooperation partnerships that may help to identify and locate perpetrators that misuse and abuse AI systems with the support of the private sector. The task is complex and needs to be achieved with the support of the technical and business community, otherwise isolated investigative and law enforcement efforts against criminals making use of AI systems will not likely succeed.

AI policy has been at the core of the discussions only in recent years. At the regional level, the European Commission has recently published a regulation proposal known as the *Digital Services Act*^29^ though this proposal has just recently been opened for consultation and it will take a few years until it is finally approved.

On April 21, 20021, the European Commission published its awaited *Regulation proposal for Artificial Intelligence Systems*.^30^ The proposal contains broad and strict rules and obligations before AI services can be put into the European market based on the assessment of different levels of risks. The regulation proposal of the European Commission also contains express prohibitions of AI practices that may contravene EU values and violate fundamental rights of citizens, and it establishes the European Artificial Intelligence Board (EIAB) as the official body that will supervise the application and enforcement of the regulation across the EU.^31^

The prospect of developing a new international convention that will regulate relevant aspects concerning the impact and development of AI systems and the intersection with the protection of fundamental rights has been proposed by the Ad-Hoc Committee on Artificial Intelligence of the Council of Europe, better known as ‘CAHAI’. The work of CAHAI will be analysed in section 5.1 of this paper.

## International instruments to counter cybercrime

At the international level, there are a number of international and regional instruments that are used to investigate “cyber dependent crime”, “cyber enabled crime” and “computer supported crime”.^32^ This paper will only focus on the analysis of three major instruments of the Council of Europe which are applicable to criminal conduct and activities concerning the use of computer and information systems, the exploitation and abuse of children and violence against women committed through information and computer systems:

- The Convention on Cybercrime better known as the *‘the Budapest Convention’*;
- The Convention on Protection of Children against Sexual Exploitation and Sexual Abuse, better known as *‘the Lanzarote Convention’*; and
- The Convention on preventing and combating violence against women and domestic violence better known as the *‘the Istanbul Convention’*.

### The Budapest Convention

The Council of Europe’s Budapest Convention on Cybercrime is the only international treaty that criminalizes conducts and typologies committed through computer and information systems. This instrument contains substantive and procedural provisions for the investigation, execution and adjudication of crimes committed through computer systems and information technologies.^33^ The Budapest Convention is mainly used as a vehicle for international cooperation to investigate and prosecute cybercrime among the now 66 State Parties, which includes many countries outside Europe.^34^

The Cybercrime Convention Committee (T-CY) which is formed by State Parties, country observers invited to accede to the Budapest Convention and ad-hoc participants is the entity responsible *inter alia* for conducting assessments of the implementation of the provisions of the Budapest Convention, as well as the adoption of opinions and recommendations regarding the interpretation and implementation of its main provisions.^35^

During the 2021 Octopus Conference on Cooperation against Cybercrime in November 2021 that marked the 20th anniversary of the Budapest Convention, the organizers announced that the Committee of Ministers of the Council of Europe approved the adoption of the *Second Additional Protocol to the Budapest Convention on enhanced cooperation and the disclosure of electronic evidence* as originally adopted by 24 the Plenary Session of the T-CY Committee in May 2021. The text of the Second Additional Protocol will be officially opened for signature among State parties to the Budapest Convention in the summer of 2022.^36^

The *Second Additional Protocol to the Budapest Convention on enhanced cooperation and the disclosure of electronic evidence* regulates *inter alia* how the information and electronic evidence - including subscriber information, traffic data and content data - may be ordered and preserved in criminal investigations among State Parties to the Budapest Convention. It provides a legal basis for disclosure of information concerning the registration of domain names from domain name registries and registrars and other key aspects concerning cross-border investigations including mutual legal assistance procedures, direct cooperation with service providers, disclosure of data in emergency situations, protection of safeguards for transborder access to data and joint investigation teams.^37^

Although, the T-CY Committee has not yet fully explored how the Budapest Convention and its first additional protocol on xenophobia and racism may be applicable in the context of technologies and systems based on AI, it is worth mentioning that the Budapest Convention was drafted with broad consideration of the principle of technological neutrality precisely because the original drafters of this instrument anticipated how the threat landscape for cybercrime would likely evolve and change in the future.^38^

The Budapest Convention contains only a minimum of definitions; however, this instrument criminalizes a number of conducts and typifies many offenses concerning computer and content related crimes that may as well be applicable to crimes committed through the use of AI systems.

During the 2018 Octopus Conference on Cooperation against Cybercrime, the Directorate General of Human Rights and Rule of Law of the Council of Europe convened a panel on *AI and Cybercrime*^39^ where representatives of the CoE presented its early activities and findings on AI policy.^40^ Although the panel presentations were more descriptive concerning the technical terminology used in the field AI at that time, some speakers highlighted and discussed some of the challenges that AI poses to law enforcement authorities like for instance the criminalization of video and document forgery and how authorities could advance the challenge to obtain and preserve electronic evidence in court.^41^

The 2021 Octopus Conference on Cooperation against Cybercrime held fully online from 16-18 November 2021 due to the COVID-19 situation, held a panel on “Artificial Intelligence, cybercrime and electronic evidence”.^42^ This panel discussed complex questions concerning criminal liability and trustworthiness of evidence of AI systems in auditing and driving automation and assistance; and other relevant aspects concerning harms and threats of misinformation and disinformation developed by AI systems and effective responses, countermeasures and technical solutions from the private sector.

AI and cybercrime are relevant aspects that need further analysis and detailed discussions among the TC-Y and State Parties to the Budapest Convention, particularly since there has been an increase of cases concerning the misuse of AI technologies by cybercriminals and as vehicles to launch cyberattacks and commit criminal offenses against individuals in the cyberspace. Questions such as who will bear the responsibility for a conduct committed through the use of algorithms and machine learning and the liability threshold among State Parties need further discussion and clarification since the regulation of criminal liability differs significantly among the legal systems of many countries, as well as to explore the development of strategic partnerships in other regions of the world to counter attacks based on AI systems.

### The Lanzarote Convention

The Council of Europe Lanzarote Convention is an international treaty that contains substantive legal measures for the protection of children from sexual violence including sexual exploitation and abuse of children online.^43^ This convention harmonizes minimum legal conducts at the domestic level to combat crimes against children and provide measures for international cooperation to counter the sexual exploitation of children. The Lanzarote Convention requires the current 48 State Parties to offer a holistic response to sexual violence against children through the “4Ps approach”: Prevention, Protection, Prosecution and Promotion of national and international cooperation.^44^ The monitoring and implementation body of the Lanzarote Convention is conducted by the Committee of the Parties, also known as the *‘Lanzarote Committee’*. This committee is formed by State Parties and it is primarily responsible for monitoring how State Parties put legislation, policies and countermeasures into practice, including organizing capacity building activities to exchange information and best practices concerning the implementation of the Lanzarote Convention across State Parties.^45^

Like, the TC-Y, the ‘Lanzarote Committee’ has not yet fully explored how the substantive and procedural criminal law provisions of the Lanzarote Convention may apply in the context of the use of AI systems for criminal related purposes, a situation that needs to be further discussed among State Parties in order to not only share and diffuse knowledge on current trends among State Parties of that treaty, but to also help identify illicit conducts and abuse and exploitation of children through AI systems, as well as an analysis of positive uses of AI technologies for the prevention of crimes concerning the protection of children online.

### The Istanbul Convention

The Istanbul Convention is another treaty of the Council of Europe the main purpose of which is to protect women against all forms of violence and to counter and eliminate all forms of violence against women including aspects of domestic violence.^46^ The Istanbul Convention consists of four main pillars: (i) prevention, (ii) protection of victims, (iii) prosecution of offenders, and (iv) implementation of comprehensive and coordinated policies to combat violence against women at all levels of government. The Istanbul Convention establishes an independent group of experts known as the GREVIO (Group of Experts on Action against Violence against Women and Domestic Violence). The GREVIO is responsible for monitoring the effective implementation of the provisions of the Istanbul Convention by the now 34 States Parties.^47^

The Istanbul Convention does not specifically contain specific provisions in the context of violence committed through the use of information technologies, however the GREVIO is currently analysing approaches to extend the application of the commission of illegal conducts through the use of computer and information systems within the national legal framework of State Parties.^48^ The GREVIO adopted during its twenty-fifth meeting on 20 October 2021, a *General Recommendation on the Digital Dimension of Violence against Women*.^49^ The Recommendation addresses *inter alia* the application of the general provisions of the Istanbul Convention in relation to conducts and crime typologies committed against women in cyberspace and proposes specific actions to take, based on the four pillars of the Istanbul Convention: prevention, protection, prosecution and coordinated policies.

As part of promoting the scope of the adopted General Recommendation, the GREVIO held a conference in Strasbourg in November 24, 2021 that featured a keynote address of the Commissioner of Human Rights of the Council of Europe and presentations of the President of the GREVIO and the Chair of the Committee of the Parties to the Istanbul Convention followed by a panel discussion with representatives of EU member states, internet industry and civil society.^50^ Among the relevant points made during the panel discussions were how the recommendation may help to advance legal and policy developments, attention of victims of current forms of cyberviolence, further international cooperation and to contribute to the general understanding of the scope of the provisions of the Istanbul Convention and other key instruments of the Council of Europe including the Budapest Convention and the Lanzarote Convention in relation to digital violence against women.^51^

The Cybercrime Convention Committee (T-CY) issued a comprehensive report titled *Mapping Study on Cyberviolence* with recommendations adopted by the TC-Y on 9 July, 2018.^52^

The mapping study developed a working definition on “cyberviolence”^53^ and described how the different forms of cyberviolence may be classified and criminalized under the Budapest-, Lanzarote- and Istanbul Conventions. According to the mapping study *“not all forms of violence are equally severe and not all of them necessarily require a criminal law solution but could be addressed with a combination of preventive, educational, protective and other measures”*. The main conclusions of the Cybercrime Convention Committee (T-CY) in the *Mapping Study on Cyberviolence* were:

(i) *the Budapest Convention and its additional Protocol on Racism and Xenophobia covers and address some types of cyberviolence;*
(ii) *the procedural powers and the provisions on international cooperation of the Budapest Convention will help to support the investigation of cyberviolence and the secure and preservation of digital evidence; and*
(iii) *the Budapest, the Istanbul and Lanzarote conventions complement each other and should promote synergies. These synergies may include raising further awareness and capacity building activities among Parties to said treaties; encourage parties to the Lanzarote and Istanbul Conventions to introduce the procedural powers contained in the Budapest Convention* (*Arts. 16-21*) *into domestic law and consider becoming parties to the Budapest Convention to facilitate international cooperation on electronic evidence in relation to crimes related to cyberviolence; encourage parties to the Budapest Convention to implement the provisions on psychological violence, stalking and sexual harassment of the Istanbul Convention, as well as the provisions on sexual exploitation and abuse of children online of the Lanzarote Convention, among others*.^54^

Cyberviolence and crimes concerning the abuse and exploitation of children online require strategic cooperation of different stakeholders. Other key institutions at the regional level like the European Commission have also explored paths on how AI systems may help to identify, categorise and remove child sexual abuse images and to minimise the exposure of human investigators to distressing images and the importance of the role of internet hotlines in facilitation the reporting process.^55^

## Ongoing work of international organizations

### Council of Europe CAHAI

The Ad-Hoc Committee on Artificial Intelligence of the Council of Europe (CAHAI)^56^ was established by the Committee of Ministers during its 1353rd meeting on 11 September 2019.^57^ The specific task of CAHAI is “to complete the feasibility study and produce the potential elements on the basis of broad multi-stakeholder consultations, of a legal framework for the development, design and application of artificial intelligence, based on the Council of Europe’s standards on human rights, democracy and the rule of law.”

The work of CAHAI is relevant because it sets forth a multi-stakeholder group where global experts may provide their views on the development of policies on AI, to forward meaningful proposals to ensure the application of international treaties and technical standards on AI and submit proposals for the creation of a future legal instrument that will regulate AI while ensuring the protection of fundamental rights, rule of law and democracy principles contained in relevant instruments of the Council of Europe, like Convention 108+, the Budapest, Lanzarote and Istanbul Conventions, among others.^58^

The work of CAHAI will impact the 47 members states and country observers of the Council of Europe, particularly state institutions including national parliamentarians and policy makers who are responsible for the implementation of international treaties into their national legal frameworks. Therefore, the inclusion and participation of relevant stakeholders from different nations will play a decisive role in the future implementation of a global treaty on AI in the coming years.

### European Parliament

The European Parliament (EP) is perhaps the most proactive legislative and policy making institution worldwide. The European Parliament has a Centre for Artificial Intelligence known as (C4AI) that was established in December 2019.^59^ The EP has Committees that analyse the impact of policy related aspects of AI in many different areas including cybersecurity, defence, predictive policing and criminal justice. The most active committee is the Special Committee on Artificial Intelligence in a Digital Age (AIDA Committee)^60^ that has organized many hearings and workshops with different experts and stakeholders on AI from different regions of the world to hear views and opinions on the *Regulation proposal for Artificial Intelligence Systems*.^61^

According to the President of the AIDA Committee, *“the use of AI in law enforcement is a political decision and not a technical one, our duty is to apply the political worldview to determine what are the allowed uses of AI and under which conditions”*.^62^

As a result of the existing dangers and risks posed by the use of AI systems across Europe, the European Parliament adopted a resolution on 6 October 2021 that calls for a permanent ban on AI systems which allow for the use of automated recognition of individuals by law enforcement in public spaces. Further, the resolution calls for a moratorium on the deployment of facial recognition systems for law enforcement purposes and a ban on predictive policing based on behavioural data and social scoring in order to ensure the protection of fundamental rights of European citizens.^63^

The Committee on Civil Liberties, Justice and Home Affairs of the European Parliament has also conducted relevant work on AI and criminal justice. On February 20, 2020, said committee conducted a public hearing on “Artificial Intelligence in Criminal Law and its use by the Police and Judicial Authorities” where relevant opinions and recommendations of experts and international organizations were discussed and presented.^64^

Further, the AIDA Committee of the European Parliament held a two-day public hearing with the AFET Committee on March 1^st^ and 4^th^ 2021. The first hearing was on “AI Diplomacy and Governance in a Global Setting: Toward Regulatory Convergence”, and the second hearing on “AI, Cybersecurity and Defence”.^65^ Many relevant aspects of AI policy were mentioned during the hearings, including the support of a transatlantic dialogue and cooperation on AI, the development of ethical frameworks and standards, the development of a shared system of norms, respect of fundamental rights, diplomacy and capacity building among others. Although, there was mention on the importance of AI for cybersecurity in the defence realm and how AI might be helpful to mitigate cyberattacks and protect critical infrastructure, there was no specific mention on how the current international treaties on cybercrime and national legal frameworks may coexist with a future treaty on AI to counter cybercrime more effectively.

The dialogue and engagement of the different committees of the European Parliament on AI policy is key for the future implementation of policies in the criminal justice area concerning the use and deployment of AI systems and applications. The European Parliament should continue to promote further dialogues and activities with other international organizations like the Council of Europe and the OECD, as well as with national parliamentarians around the world to help them understand the dimensions and implications of creating regulations and policies on AI to specifically counter cybercrime.

### The UN Interregional Crime and Justice Research Institute (UNICRI) Centre for Artificial Intelligence and Robotics

The Centre for Artificial Intelligence and Robotics of the United Nations Interregional Crime and Justice Research Institute (UNICRI), a research arm of the United Nations is very active in the organization of workshops and information and reports to demystify the world of robotics and AI and to facilitate an in-depth understanding of the crimes and threats conducted through AI systems among law enforcement officers, policy makers, practitioners, academia and civil society. UNICRI and INTERPOL drafted the report “*Artificial Intelligence and Robotics for Law Enforcement”*^66^ in 2019 that draws upon the discussions of a workshop held in Singapore in July 2018. Among the main findings of UNICRI and INTERPOL’s report are:

*“AI and Robotics are new concepts for law enforcement and there are expertise gaps that should be filled to avoid law enforcement falling behind.”*

*“Some countries have explored further than others and a variety of AI techniques are materializing according to different law enforcement authorities. There is, however, a need for greater international coordination on this issue.”*

The mandate of the Centre for Artificial Intelligence and Robotics of UNICRI is quite broad. It covers policy related aspects of AI in the field of criminal justice including areas such as cybersecurity, autonomous weapons, self-driving vehicles and autonomous patrol systems. UNCRI organizes every year the *Global Meeting on Artificial Intelligence for Law Enforcement*, an event that discusses relevant developments on AI with experts and stakeholders from different sectors and countries to enhance and improve the capabilities for law enforcement authorities and the criminal justice system in the use and deployment of AI technologies.^67^

The Centre for Artificial Intelligence and Robotics of UNICRI is currently working with a group of experts from INTERPOL, the European Commission and other relevant institutions and stakeholders in the development of a *Toolkit for Responsible AI Innovation in Law Enforcement*. The toolkit will provide and facilitate practical guidance for law enforcement agencies around the world on the use of AI in a trustworthy, lawful and responsible manner. The toolkit addresses practical insights, use cases, principles, recommendations, best practices and resources which will help to support law enforcement agencies around the world to use AI technologies and applications.^68^

## Conclusion

The use of AI systems across different sectors is an ongoing trend, and this includes authorities of the criminal justice system which have realized the benefits and advantages of using this technology. National law enforcement authorities involved in the investigation of cybercrime are not yet fully prepared to deal with the technical and legal dimensions of AI when used for disruptive or malicious purposes. Further, there is no yet sufficient evidence to justify whether law enforcement authorities around the world are well equipped and trained to gather cross-border evidence to conduct national investigations where an AI system was involved in the commission or perpetration of an illicit conduct.

Second, the coordination and cooperation with service providers and companies that manage and operate AI systems and services is crucial to help determine its abuse and misuse by perpetrators. However, these tasks bring a number of technical and legal challenges, since most AI systems rely on an internet connection to function where oftentimes subscriber and traffic data is needed to conduct an investigation. Therefore, global service providers will also have an important role to play in the possible identification and location of cybercriminals, a situation that needs well-coordinated efforts, measures and responses based on international treaties and national laws between law enforcement authorities and private sector entities. The need for further strategic partnerships to counter cybercrime is more important than ever.

The future work of international organizations like UNICRI, the Council of Europe through CAHAI and the T-CY Committee of the Budapest Convention will be very relevant for policy makers and law enforcement authorities for the correct guidance in the implementation of future national policies on AI. The CAHAI may fill up the missing discussions in international fora concerning AI to specifically counter cybercrime based on the current standards of the Council of Europe like the Budapest Convention, the Lanzarote Convention and the Istanbul Convention, as well as the emerging practices of members states to specifically counter cyber enable crimes.

The creation of national taskforces on cybercrime (composed of law enforcement authorities, representatives of the judiciary, AI technology developers and global service providers) may serve as a relevant vehicle to coordinate and tackle illicit conducts concerning the misuse and abuse of AI technologies. These taskforces may be articulated in the context of the national strategies on AI and should be linked to the tasks of the criminal justice authorities to specifically counter cybercrime.

## Footnotes

1 Burgess, Matt, “Police built an AI to predict violent crime. It was seriously flawed”, WIRED, August 6, 2020, available at: https://www.wired.co.uk/article/police-violence-prediction-ndas.

2 European Commission, “Liability for Artificial Intelligence and other emerging digital technologies”, Report from the Experts Group on Liability and New Technologies-New Technologies Formation, European Union 2019, available at: https://digital-strategy.ec.europa.eu/en/policies/european-approach-artificial-intelligence. See also: European Parliament Research Service (EPRS), “The European added value of a common EU approach to liability rules and insurance for connected and autonomous vehicles” Study published by the European Added Value Unit, February 2018, available at: https://www.europarl.europa.eu/RegData/etudes/STUD/2018/615635/EPRS_STU(2018)615635_EN.pdf.

3 MIT Technology Review, “Transforming the Energy Industry with AI”, January 21, 2021, available at: https://www.technologyreview.com/2021/01/21/1016460/transforming-the-energy-industry-with-ai/.

4 World Health Organization (WHO), “WHO reports fivefold increase in cyberattacks, urges vigilance”, April 23, 2020, available at: https://www.who.int/news/item/23-04-2020-who-reports-fivefold-increase-in-cyber-attacks-urges-vigilance.

5 The New York Times, “Cyber Attack Suspected in German Woman’s Death”, September 18, 2020, available at: https://www.nytimes.com/2020/09/18/world/europe/cyber-attack-germany-ransomeware-death.html.

6 Supply Chain, “Lessons Learned from the Vaccine Supply Chain Attack”, January 16, 2021, available at: https://www.supplychaindigital.com/supply-chain-risk-management/lessons-learned-vaccine-supply-chain-attack.

7 Prakarsh and Riya Khanna, “Artificial Intelligence and Cybercrime- A curate’s Egg”, Medium, June 14, 2020, available at: https://medium.com/the-%C3%B3pinion/artificial-intelligence-and-cybercrime-a-curates-egg-2dbaee833be1.

8 INTSIGHTS, “The Dark Side of Latin America: Cryptocurrency, Cartels, Carding and the Rise of Cybercrime”, p.6, available at: https://wow.intsights.com/rs/071-ZWD-900/images/Dark%20Side%20of%20Latin%20America.pdf. See also, “The Next, El Chapo is Coming for your Smartphone”, June 26, 2020, available at: https://www.ozy.com/the-new-and-the-next/the-next-el-chapo-might-strike-your-smartphone-and-bank/273903/.

9 Malwarebytes Lab, “When Artificial Intelligence goes awry: separating science fiction from fact”, without publication date, available at: https://resources.malwarebytes.com/files/2019/06/Labs-Report-AI-gone-awry.pdf.

10 SIEMENS Energy, “Managed Detection and Response Service”, 2020, available at: https://assets.siemens-energy.com/siemens/assets/api/uuid:a95b9cd3-9f4d-4a54-8c43-77fbdb6f418f/mdr-white-paper-double-sided-200930.pdf.

11 POLITICO, “Automated racism: How tech can entrench bias”, March 2, 2021, available at: https://www.politico.eu/article/automated-racism-how-tech-can-entrench-bias/.

12 For a discussion on discrimination caused by algorithmic decision making on AI, see ZUIDERVEEN BORGESIUS, Frederik, “Discrimination, Artificial Intelligence and Algorithmic decision making”. Paper published by the Directorate General of Democracy of the Council of Europe, 2018, available at: https://rm.coe.int/discrimination-artificial-intelligence-and-algorithmic-decision-making/1680925d73.

13 See the Special Report on Facial Recognition of the Center for AI and Digital Policy (CAIDP) that contains a summary of key references on this topic contained in the *2020 Report on Artificial Intelligence and Democratic Values*/ *The AI Social Contract Index 2020* prepared by CAIDP, December 2020, available at: https://caidp.dukakis.org/aisci-2020/.

14 In October 2021, the European Parliament adopted a resolution to ban the use facial recognition technologies in public spaces by law enforcement authorities to ensure the protection of fundamental rights. See European Parliament, “Use of Artificial Intelligence by the police: MEPs oppose mass surveillance”. LIBE Plenary Session press release, October 6, 2021, available at: https://www.europarl.europa.eu/news/en/press-room/20210930IPR13925/use-of-artificial-intelligence-by-the-police-meps-oppose-mass-surveillance.

15 BBC, “What are ‘bots’ and how can they spread fake news, available at: https://www.bbc.co.uk/bitesize/articles/zjhg47h.

16 FORBES, *“Fake News is Rampant, Here is How Artificial Intelligence Can Help”*, January 21, 2021, available at: https://www.forbes.com/sites/bernardmarr/2021/01/25/fake-news-is-rampant-here-is-how-artificial-intelligence-can-help/?sh=17a6616e48e4.

17 European Commission, “Tackling online disinformation”, 18 January 2021, available at: https://ec.europa.eu/digital-single-market/en/tackling-online-disinformation. For a general review of policy implications in the UK concerning the use of AI and content moderation, see Cambridge Consultants, *“Use of AI in Online Content Moderation”*. 2019 Report produced on behalf of OFCOM, available at: https://www.ofcom.org.uk/__data/assets/pdf_file/0028/157249/cambridge-consultants-ai-content-moderation.pdf.

18 Deepfakes are based on AI deep learning algorithms, an area of machine learning that applies neural net simulation to massive data sets to create fakes videos of real people. Deepfakes are trained algorithms that allows the recognition of data patterns, as well as human facial movement and expressions and can match voices that can imitate the real voice and gestures of an individual. See: European Parliamentary Research Service, “What if deepfakes made us doubt everything we see and hear (Science and Technology podcast], available at: https://epthinktank.eu/2021/09/08/what-if-deepfakes-made-us-doubt-everything-we-see-and-hear/. Like, many technologies, deepfakes can be used as a tool for criminal related purposes such as fraud, extortion, psychological violence and discrimination against women and minors, see: MIT Technology Review, “A deepfake bot is being used to “undress” underage girls”, October 20, 2020, available at: https://bit.ly/3qj1qWx.

19 For specific information regarding the work of the US government to counter the use of deepfakes, see CNN, “*Inside the Pentagon’s race against deepfake videos”*, available at: https://bit.ly/38aEqCShttps://edition.cnn.com/interactive/2019/01/business/pentagons-race-against-deepfakes/.

20 EURACTIV, “EU police recommend new online ‘screening tech’ to catch deepfakes”, November 20, 2020, available at: https://www.euractiv.com/section/digital/news/eu-police-recommend-new-online-screening-tech-to-catch-deepfakes/.

21 The Verge, “Watch Jordan Peele use AI to make Barack Obama deliver a PSA about fake news”, April 17, 2018, available at: https://www.theverge.com/tldr/2018/4/17/17247334/ai-fake-news-video-barack-obama-jordan-peele-buzzfeed.

22 Wall Street Journal, “Fraudsters Use AI to Mimic CEO’s Voice in Unusual Cybercrime Case”, August 30, 2019, available at: https://www.wsj.com/articles/fraudsters-use-ai-to-mimic-ceos-voice-in-unusual-cybercrime-case-11567157402.

23 GIZMODO, “Bank Robbers in the Middle East Reportedly ‘Cloned’ Someone’s Voice to Assist with $35 Million Heist”, October 14, 2021, available at: https://gizmodo.com/bank-robbers-in-the-middle-east-reportedly-cloned-someo-1847863805.

24 The EC3 of Europol has developed good capacities and practice with other countries in the deployment of joint investigation teams to counter organized crime, including cybercrime. See the section on Join Investigation Team of Europol at: https://www.europol.europa.eu/activities-services/joint-investigation-teams.

25 INTERPOL (EC3), “Internet Crime Assessment Report 2020” (IOCTA 2020 Report), p. 18, available at: https://www.europol.europa.eu/activities-services/main-reports/internet-organised-crime-threat-assessment-iocta-2020. The Internet Crime Assessment Report 2021 (IOCTA 2021 Report) was published on 11 November 2021. The report of this year does not actually make any novel references to misuse and abuse of AI systems for criminal purposes, available at: https://www.europol.europa.eu/activities-services/main-reports/internet-organised-crime-threat-assessment-iocta-2021.

26 IOCTA 2020 Report, *Op. cit*. note 25, p. 18.

27 Trend Micro Research, EUROPOL EC3 and UN Interregional Crime and Justice Research Institute (UNICRI), *Malicious Uses and Abuses of Artificial Intelligence*, 19 November 2020, available at: https://www.europol.europa.eu/publications-documents/malicious-uses-and-abuses-of-artificial-intelligence.

28 Trend Micro Research, EUROPOL EC3 and UN Interregional Crime and Justice Research Institute (UNICRI), *Malicious Uses and Abuses of Artificial Intelligence*, 19 November 2020, available at: https://www.europol.europa.eu/publications-documents/malicious-uses-and-abuses-of-artificial-intelligence.

29 This report was also presented in a workshop on cybercrime, e-evidence and artificial intelligence during the 2021 Octopus Conference on Cooperation against Cybercrime organized by the Council of Europe on November 17, 2021 where the representatives of each organization highlighted the main aspects and features of the report, including current trends and concrete examples of misuse of AI technologies. The presentation is available at: https://rm.coe.int/edoc-1193149-v1-coe-ai-ppt/1680a4892f. The Digital Services Act establishes new rules and requirements for intermediary service providers which includes hosting providers and online platforms. This regulation covers inter alia rules on liability for online intermediary service platforms, establishes internal complaint handling systems and implement measures against online legal content. The Digital Services Act is currently a draft proposal under discussion between the European Parliament and the Council of the EU and it may take some years until it is finally approved, available at: https://digital-strategy.ec.europa.eu/en/policies/digital-services-act-package.

30 See Proposal for a Regulation of the European Parliament and the Council laying down harmonized rules on Artificial Intelligence (Artificial Intelligence Act) and Amending Certain Union Legislative Acts, Brussels 21.4.2021, available at: https://eur-lex.europa.eu/legal-content/EN/TXT/HTML/?uri=CELEX:52021PC0206&from=EN.

31 See: European Commission, “Europe fit for the Digital Age: Commission proposes new rules and actions for excellence and trust in Artificial Intelligence”, Brussels, April 21, 2021, available at: https://ec.europa.eu/commission/presscorner/detail/en/ip_21_1682. See also the website of the European Commission that explains the approach of the EC on AI and the relevant milestones in this area, available at: https://digital-strategy.ec.europa.eu/en/policies/european-approach-artificial-intelligence.

32 Among those instruments are: (i) The United Convention against Organized Crime and its Protocols (*Palermo Convention*); (ii) The Council of Europe Convention on Cybercrime (*Budapest Convention*) and its Additional Protocol concerning the criminalisation of acts of a racist and xenophobic nature committed through computer systems; (iii) The Council of Europe Convention on Protection of Children against Sexual Exploitation and Sexual Abuse (*Lanzarote Convention*); (iv) The African Union Convention on Cyber Security and Personal Data Protection (*Malabo Convention*); (v) Directive 2013/40/UE on attacks against information systems; (vi) Directive 2011/92/UE on combating the sexual abuse and exploitation of children and child pornography, among others.

33 The Budapest Convention requires that Party States amend their substantive and procedural criminal legislation to make it consistent with the substantive and procedural criminal law provisions of that treaty. Considering that cybercrime has a transnational dimension, the Budapest Convention also requires that countries implement international cooperation measures either to supplement or complement the existing ones, particularly when a country does not have mutual assistance and cooperation treaties in criminal matters in place, as well as to equip investigative and law enforcement authorities with the necessary tools and procedural mechanisms to conduct cybercrime investigations including measures concerning: (i) expedited preservation of stored computer data, (ii) disclosure of preserved traffic data, (iii) mutual assistance measures regarding access to stored computer data, (iv) trans-border access to stored computer data, (v) mutual assistance regarding real-time collection of traffic data, (vi) mutual assistance regarding the interception of content data, and the (vii) creation of a network or point of contact 24/7 to centralize investigations and procedures related to requests for data and mutual assistance concerning cybercrime investigations with other 27/7 points of contact.

34 See the Budapest Convention Chart of Signatures and Ratifications at: https://www.coe.int/en/web/conventions/full-list/-/conventions/treaty/185/signatures?p_auth=yUQgCmNc.

35 Cybercrime Convention Committee, “T-CY Rules of Procedure. As revised by T-CY on 16 October 2020”, Strasbourg, 16 October 2020, available at: https://rm.coe.int/t-cy-rules-of-procedure/1680a00f34.

36 Council of Europe, “Second Additional Protocol to the Budapest Convention adopted by the Committee of Ministers of the Council of Europe”, Strasbourg, 17 November 2021, available at: https://www.coe.int/en/web/cybercrime/-/second-additional-protocol-to-the-cybercrime-convention-adopted-by-the-committee-of-ministers-of-the-council-of-europe.

37 See the text of the Explanatory Report of the Second Additional Protocol to the Budapest Convention drafted by Cybercrime Convention Committee (T-CY) at: https://search.coe.int/cm/pages/result_details.aspx?objectid=0900001680a48e4b.

38 See the Explanatory Report to the Convention on Cybercrime at: https://rm.coe.int/CoERMPublicCommonSearchServices/DisplayDCTMContent?documentId=09000016800cce5b.

39 The Conference program of the 2018 Octopus conference on cooperation against cybercrime is available at: https://rm.coe.int/3021-90-octo18-prog/16808c2b04.

40 See: Activities of the Council of Europe on Artificial Intelligence (AI), 9 May, 2018, available at: https://rm.coe.int/cdmsi-2018-misc8-list-ai-projects-9may2018/16808b4eac.

41 See the presentations of this panel at the Plenary Closing session of the 2018 Octopus Conference, available at: https://www.coe.int/en/web/cybercrime/resources-octopus-2018.

42 The presentation and materials of this panel are available at: https://www.coe.int/en/web/cybercrime/workshop-cybercrime-e-evidence-and-artificial-intelligence.

43 The Lanzarote Convention entered in force on 1 July 2010, available at: https://www.coe.int/en/web/conventions/full-list/-/conventions/treaty/201/signatures. Among the conducts that the Lanzarote Convention requires Sates parties to criminalize are: (i) Child sexual abuse; (ii) sexual exploitation through prostitution; (iii) child sexual abuse material; (iv) exploitation of a child in sexual performances; (v) corruption of children, and (vi) solicitation of children for sexual purposes.

44 See the Booklet of the Lanzarote Convention, available at: https://rm.coe.int/lanzarote-convention-a-global-tool-to-protect-children-from-sexual-vio/16809fed1d.

45 The Rules of procedure, adopted documents, activity reports and the Meetings of the ‘Lanzarote Committee’ are available at: https://www.coe.int/en/web/children/lanzarote-committee#{%2212441908%22:[].

46 The Istanbul Convention entered into force on 1 August 2014 and it has been ratified by 34 countries. See the chart of signatures and ratifications at: https://www.coe.int/en/web/conventions/full-list/-/conventions/treaty/210/signatures?p_auth=OwhAGtPd.

47 The Rules of procedure and adopted documents of the GREVIO are available at: https://www.coe.int/en/web/istanbul-convention/grevio.

48 See the presentations of the webinar, “Cyberviolence against Women” organized by the CyberEast Project of the Council of Europe, 12 November, 2020, available at: https://www.coe.int/en/web/cybercrime/cyberviolence-against-women.

49 The Text of the GREVIO General Recommendation No. 1 on the digital dimension of violence against women adopted on 20 October 2021 is available at: https://rm.coe.int/grevio-rec-no-on-digital-violence-against-women/1680a49147.

50 Council of Europe, “Launch Event: Combating violence against women in a digital age-utilizing the Istanbul Convention”, 24 November 2021, available at: https://www.coe.int/en/web/istanbul-convention/launching-event-of-grevio-s-first-general-recommendation-on-the-digital-dimension-of-violence-against-women.

51 Council of Europe Media Release, “New Council of Europe Recommendation tackles the ‘digital dimension” of violence against women and girls”, Strasbourg, 24 November, 2021, available at: https://search.coe.int/directorate_of_communications/Pages/result_details.aspx?ObjectId=0900001680a4a67b.

52 Council of Europe Cybercrime Convention Committee (TC-Y), “Mapping Study on Cybercrime” with recommendations adopted by the TC-Y on 9 July 2018, available at: https://rm.coe.int/t-cy-2017-10-cbg-study-provisional/16808c4914.

53 The definition is an adaptation of the definition of violence against women contained in Art. 3 of the Istanbul Convention to the cyber context as follows: “*Cyberviolence is the use of computer systems to cause, facilitate, or threaten violence against individuals that results in, or is likely to result in, physical, sexual, psychological or economic harm or suffering and may include the exploitation of the individual’s circumstances, characteristics or vulnerabilities”*.

54 “Mapping Study on Cybercrime”, *Op. cit*. note 52, pp. 42-43.

55 European Commission, “Exploring potential of AI in fight against child online abuse”, Event report 11 June 2020, available at: https://ec.europa.eu/digital-single-market/en/news/exploring-potential-ai-fight-against-child-online-abuse.

56 CAHAI’s composition consist of three main groups composed of up to 20 experts appointed by Members States, as well as observers and participants. The mandate of the Policy Development Group (CAHAI-PDG) is the development of the feasibility study of a legal framework on artificial intelligence applications, building upon the mapping work already undertaken by the CAHAI and to prepare key findings and proposals on policy and other measures, to ensure that international standards and international legal instruments in this area are up-to-date and effective and prepare proposals for a specific legal instrument regulating artificial intelligence. The Consultation and Outreach Group (CAHAI-COG) is responsible for taking stock of the analysis undertaken by the Secretariat of responses to online consultations and analysis of ongoing developments and reports which are directly relevant for CAHAI’s working groups’ tasks. The Legal Frameworks Group (CAHAI-LFG) is responsible for the preparation of key findings and proposals on possible elements and provisions of a legal framework with a view to draft legal instruments, for consideration and approval by the CAHAI, taking into account the scope of existing legal instruments applicable to artificial intelligence and policy options set out in the feasibility study approved by the CAHAI. Further info on the composition of CAHAI working groups, the plenary meetings and the documents issued by the three working groups is available at: https://www.coe.int/en/web/artificial-intelligence/cahai.

57 The terms of reference of CAHAI are available at: https://search.coe.int/cm/Pages/result_details.aspx?ObjectId=09000016809737a1.

58 The Final Virtual Plenary Meeting of CAHAI from 30.11.2021 to 02.12.2021 will facilitate meaningful discussions towards the adoption of a document outlining the possible elements of a legal framework on AI, which may include binding and non-binding standards based on the Council of Europe’s standards on human rights, democracy and rule of law. See Council of Europe, “The CAHAI to hold its final meeting”, Strasbourg, 24 November 2021, available at: https://www.coe.int/en/web/artificial-intelligence/-/cahai-to-hold-its-final-meeting.

59 European Parliament, “STOA Centre for Artificial Intelligence (C4AI)”. The C4AI produces studies, organises public events and acts as a platform for dialogue and information exchange and coordinate its efforts and influence global AI standard-setting, available at: https://www.europarl.europa.eu/stoa/en/centre-for-AI.

60 The AIDA Committee website is available at: https://www.europarl.europa.eu/committees/en/aida/home/highlights.

61 See *supra* note 30.

62 See Dragos Tudorache Plenary Speech on Artificial Intelligence of 4 October 2021, available at: https://www.youtube.com/watch?v=V9y5gt39AD0.

63 European Parliament News, “Use of artificial intelligence by the police: MEPs oppose mass surveillance”. Press release of the Plenary Session, October 6, 2021, available at: https://www.europarl.europa.eu/news/en/press-room/20210930IPR13925/use-of-artificial-intelligence-by-the-police-meps-oppose-mass-surveillance and Eurocadres, “European Parliament adopts resolution on the use of AI in law enforcement”, October 6, 2021, available at: https://www.eurocadres.eu/news/european-parliament-adopts-resolution-on-the-use-of-ai-in-law-enforcement/.

64 European Parliament. “MEPs to look into Artificial Intelligence in criminal law on Thursday”, February 18, 2020, available at: https://www.europarl.europa.eu/news/en/press-room/20200217IPR72718/meps-to-look-into-artificial-intelligence-in-criminal-law-on-thursday.

65 European Parliament, Special Committee on Artificial Intelligence in a Digital Age (AIDA), “Joint hearing on the external policy dimension of AI”, March 1^st^ and 4^th^ 2021, available at: https://www.europarl.europa.eu/meetdocs/2014_2019/plmrep/COMMITTEES/AIDA/DV/2021/03-01/Final_Programme_externalpolicydimensionofAI_V26FEB_EN.pdf.

66 UNICRI and INTERPOL, “*Artificial Intelligence and Robotics for Law Enforcement”*, 2019, available at: https://issuu.com/unicri/docs/artificial_intelligence_robotics_la/4?ff.

67 UNCRI, “2^nd^ INTERPOL, UNICRI Global Meeting on Artificial Intelligence for Law Enforcement”, Singapore, July 3, 2019, available at: http://www.unicri.it/news/article/ai_unicri_interpol_law_enforcement.

68 UNICRI, “The European Commission provides support to UNICRI for the Development of the Toolkit for Responsible AI Innovation in Law Enforcement”, The Hague, Monday November 1, 2021, available at: http://www.unicri.it/index.php/News/EC-UNICRI-agreement-toolkit-responsible-AI.

